# IL-13 Genetic Susceptibility to Bullous Pemphigoid: A Potential Target for Treatment and a Prognostic Marker

**DOI:** 10.3389/fimmu.2022.824110

**Published:** 2022-01-24

**Authors:** Yiman Wang, Xuming Mao, Yangchun Liu, Yuyan Yang, Hongzhong Jin, Li Li

**Affiliations:** ^1^ Department of Dermatology, State Key Laboratory of Complex Severe and Rare Diseases, Peking Union Medical College Hospital, Chinese Academy of Medical Science and Peking Union Medical College, National Clinical Research Center for Dermatologic and Immunologic Diseases, Beijing, China; ^2^ Department of Dermatology, University of Pennsylvania, Philadelphia, PA, United States

**Keywords:** bullous pemphigod, cytokine, SNP, IL-13, recurrence, gender

## Abstract

**Background:**

Bullous pemphigoid (BP) is a senile chronic autoimmune bullous skin disease with a high relapse rate, which significantly impairs patients’ quality of life and contributes to disease mortality. This observational case-control study explores the gene polymorphisms of cytokines and their clinical significance in Chinese patients with BP.

**Methods:**

IL-1α (rs1800587), IL-1β (rs16944, rs1143627, rs1143634), IL-4 (rs2243250), IL-6 (rs1800795), IL-10 (rs1800896, rs1800871, rs1800872), IL-13 (rs1800925, rs20541), TNF-α (rs1799964, rs1800630, rs1799724, rs361525), IFN-γ (rs1799964, rs1800630, rs361525, rs1800629, rs4248160, rs1800750), and TGF-β1 (rs2317130, rs1800469, rs4803457) genes were genotyped in the healthy controls and BP patients, respectively. Expression of these cytokines in serum was measured. Medical profiles of patients, including baseline characteristics and prognosis, were statistically analyzed.

**Results:**

We found that IL-1 β and IL-13 concentrations were higher in the BP patients’ sera compared to those in the controls. For IL-13, significant differences were found in the nucleotide ratio/genotype/haploid frequency/haplotype, respectively. IL-13 (rs20541, rs1800925) is related to gender, and the IL-13 genotype was significantly associated with recurrence.

**Conclusions:**

BP is associated with IL-13 gene polymorphism and IL-13 concentration is elevated in blood circulation in patients with BP. Our results support that IL-13 is relevant in the pathogenesis of BP, suggesting that IL-13 could potentially represent a promising target for BP therapy and a prognostic marker.

## Introduction

Bullous pemphigoid (BP) is a chronic autoimmune skin disease with severe pruritus most commonly identified in the elderly. It is characterized by urticarial, erythematous, or painful blisters in the skin and circulating autoantibodies against the hemidesmosomal molecules, BP180 and BP230, located in the basement membrane zone and basal keratinocytes of the skin (1). BP represents the most common autoimmune blistering disease, albeit rare, with a reported incidence ranging from approximately 4.5 to 14 per million per year (2, 3). Moreover, the prevalence rate has been increasing markedly in the past years (4, 5).

Histopathology of the BP lesion has revealed subepidermal blisters, typically with neutrophilic and eosinophilic infiltration. Although the exact mechanisms remain to be elucidated, it is well established that BP is triggered by the autoantibodies against BP180 (6) potentially *via* three pathways (7). First, direct binding of the antibodies to an extracellular noncollagenous domain (NC16A) of BP180 can cause a conformation change, thus preventing the natural assembly of hemidesmosomes. Second, binding of the antibodies may lead to signaling activation and hemidesmosomal disassembly in the basal keratinocytes, playing a significant role in pathophysiology of BP. Alternatively, binding of the autoreactive IgG1 or 4 recruits and activates neutrophils and eosinophils to the basement membrane and activation of complements, which can release and activate proteolytic enzymes including the neutrophil elastase (NE) and matrix metalloproterinase-9 to degrade the extracellular portion of BP180 (8, 9). A body of accumulating evidence has supported that the BP autoreactive antibodies mediated a cascade of inflammatory responses that could play a critical role in BP disease pathogenesis. These include activation of complements, aggregation of neutrophils and eosinophils, increase of cytokine expression, and secretion of proteolytic enzymes (1, 5, 10, 11).

Multiple cytokines appear indispensable in developing the local immune and inflammatory responses primarily by recruiting inflammatory cells and activating resident cells in BP pathogenesis (12) and regulating the humoral or cellular immune response (13). Significantly elevated levels of cytokines were observed in the blister fluid (14–16) and the sera from BP patients (17, 18). These cytokines function as proinflammatory cytokines that further stimulate cytokine expression through a series of signaling cascades (19). Immunohistochemical and *in situ* hybridization studies have shown a significant expression of Th2 cytokines such as IL-4, IL-5, and IL-13 in the mononuclear cells in the BP lesional or perilesional skin (20, 21). More recently, the concentration of some cytokines has been correlated with BP severity and disease activity (20, 22, 23). Given their importance in BP disease, these cytokines potentially serve as promising targets for developing novel, effective therapeutic strategies for BP.

Cytokine gene polymorphisms or variations may affect cytokine functions by regulating cytokine protein expression and cytokine release, modulating susceptibility to autoimmune diseases. Interestingly, gene polymorphisms of various cytokines have been reported in some other autoimmune diseases (24–26). However, the significance of cytokine polymorphisms in BP disease has not been adequately investigated. In a previous Chinese cohort study, a polymorphism in IL-1β was identified in female patients (27). Recently, some IL-13 variation has been identified as a protective marker from BP in Iranian patients (28). In order to explore the gene polymorphisms of cytokines and their clinical significance in Chinese patients with BP, we performed a retrospective case-control association study to elucidate genetic polymorphisms of various cytokines and their relationship with serum cytokine level, clinical manifestation, and prognosis of patients. Our goal was to identify the key cytokines most relevant to BP and explore the relationship of patients’ characteristics with gene polymorphisms of these cytokines.

## Results

### Characteristics of Bullous Pemphigoid Patients

The 61 BP patients tested for SNP consisted of 30 males and 31 females, ranging from 31 to 89 years, with a mean age of 69.36 ± 15.28. The 56 patients tested for cytokine serum level consisted of 27 males and 29 females, ranging from 31 to 96 years, with a mean age of 72.09 ± 15.24 years. There was no significant difference between patients and controls regarding age and gender in SNP and serum level analysis (P > 0.05). The frequencies of TGF-β1 (rs4803457) SNP in both patient and control groups deviated markedly from Hardy–Weinberg equilibrium and were excluded from further analysis, while the genotype frequencies of all other polymorphisms were within the equilibrium.

### Allele and Genotype Frequencies of Cytokines

Gene variations of IL-1α, IL-1β, IL-4, IL-6, IL-10, IL-13, TNF-α, IFN-γ, and TGF-β1 were analyzed in BP patients compared to healthy controls to clarify whether these polymorphisms are associated with BP (**Supplementary Table 1**). We found GG is the most common genotype of IL-13 in healthy control, while the AG and AA genotypes are considered as variants. In BP patients, the proportion of IL-13 rs20541 AG genotype was significantly higher (P=0.003). The proportion of IFN - γ rs2430561 TA or TT was much higher than that of the control group. For the allele of IL-13 rs1800925, the T ratio was higher than that of the control group, and the C ratio was lower than that of the control group. For the IL-13 rs20541 allele, the G ratio was lower, whereas the A ratio was higher than that of the control group (**Table 1**).

**Table 1:** Comparison of the genotype and allele frequencies (gene polymorphisms) in the BP and control patients.

Gene	Genotype/Allele	Patients	Control	χ2/OR (95% CI)	P-Value^1^
IL-13 rs1800925	CC	41	65	3.1	0.212
	CT	16	16		
	TT	3	1		
	C Allele	98	146	0.549 (0.280-1.077)	0.078
	T Allele	22	18		
IL-13 rs20541	GG	27	58	11.85	0.003**
	AG	29	18		
	AA	4	9		
	G Allele	83	134	0.603 (0.353-1.028)	0.062
	A Allele	37	36		
IFN – γ rs2430561	TT	48	63	5.119	0.077
	AT	10	20		
	AA	3	0		
	T Allele	106	146	0.908 (0.449-1.834)	0.787
	A Allele	16	20		

^1^ **denotes P<0.01.

The frequencies of genotypes or alleles for IL-13 and INF-γ were listed in the table. The difference was compared using the Chi-square (χ2) test.

### Linkage Disequilibrium and Haplotype Frequencies

Linkage disequilibrium (LD) was performed to investigate the relationship between polymorphisms in chromosome (chr) 2, chr5, chr1, chr6, chr12, chr19 among all cytokines (**Supplementary Figure 1**). We found that rs1800871 and rs1800872 (D’=1, r2 = 1) in IL-10 are in complete linkage disequilibrium. Notably, rs16944 and rs1143627 in IL-1 (D’=1, r2 = 0.985), and rs1800630 and rs1799964 in TNF-α (D’=1, r2 = 0.759), rs2069718 and rs2430561 in IFN-γ (D’=1, r2 = 0.935), and rs2317130 and rs1800469 in TGF-β1 (D’=0.985, r2 = 0.957) have strong linkage disequilibrium. Linkage disequilibrium also exists in rs2069718 and rs2069705 in IFN-γ (D’=1, r2 = 0.501), and rs2317130 and rs4803457 in TGF-β1 (D’=1, r2 = 0.333). However, no LD is found in chr5 where IL-13 and IL-4 locate.

Haplotype frequencies were compared accordingly (**Supplementary Table 2**). Interestingly, the haploid frequency of IL-13, IL-4 (rs1800925, rs20541, rs2243250) in chr5 was different between the patient and control group, and the proportion of C A C haplotype was more [P=0.05, OR & 95%CI: 47.626 (3.156-718.771)], though not significantly, in the BP group (**Table 2**).

**Table 2:** Haplotype patterns with their frequencies in the population.

Haplotype	Case (Frequency)	Control (Frequency)	χ2	*P*-value^1^	Odds Ratio [95%CI]
C A C	3.55 (0.030)	0.10 (0.001)	3.833	0.050*	47.626 (3.156-718.771)
C A T	16.18 (0.137)	16.12 (0.101)	0.870	0.351	1.417 (0.680-2.956)
C G C	17.67 (0.150)	31.26 (0.195)	0.979	0.322	0.725 (0.383-1.373)
C G T	58.61 (0.497)	94.52 (0.591)	2.450	0.118	0.682 (0.423-1.102)
T A C	0.78 (0.007)	2.54 (0.016)	0.294	0.588	0.415 (0.052-3.311)
T A T	16.49 (0.140)	12.24 (0.077)	2.925	0.087	1.960 (0.897-4.281)
T G T	4.72 (0.040)	3.12 (0.019)	1.044	0.307	2.097 (0.492-8.947)
Global haplotype association p-value					0.080

^1^ *denotes P=0.05.

The frequencies of the haplotypes (IL-4 (rs2243250) and IL-13 (rs1800925, rs20541)) in the BP (Case) and control patients (Control) were compared using the Chi-square (χ2) test. The P values and odds ratios with a 95% confidence (95%CI) were calculated. A noticeable difference was found in the CAC(P=0.050) and TAT(P=0.087) haplotypes, respectively.

### Serum Concentration of Cytokines in Patients and Its Relationship With Genotypes

**Figure 1:**
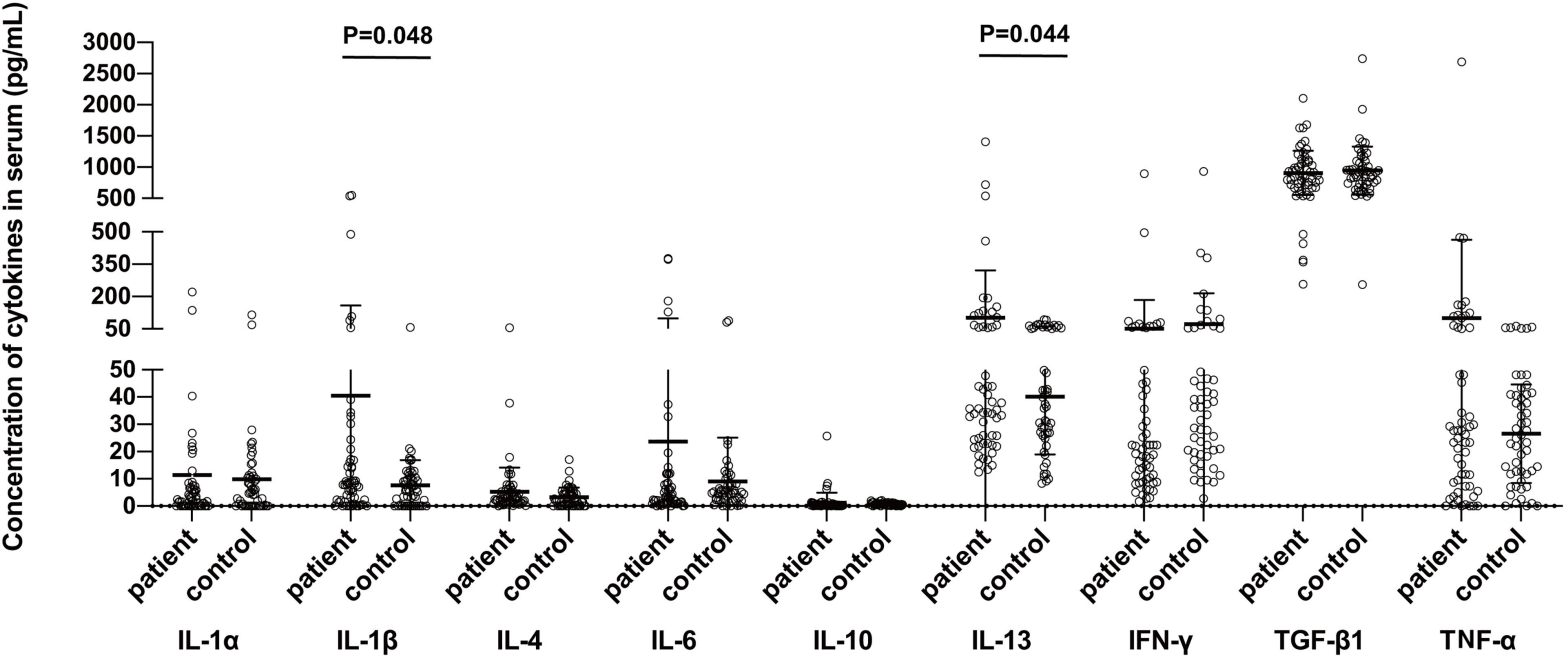
Comparison of serum cytokine levels between bullous pemphigoid patients and control groups. The protein concentrations were measured in the BP patients and healthy control subjects using the commercially available ELISA kits. Statistical analysis revealed a significant difference in the concentrations of IL-1β (P=0.048) and IL-13 (P=0.044) between the patient and control groups, respectively.

The serum concentrations of IL-1 β ((P=0.048) and IL-13 (P=0.044), but not of the IL-1α, IL-4, IL-6, IL-10, IFN-γ, TNF-α, or TGF-β, were found significantly higher in the sera of BP patients as compared to those of the controls (**Figure 1**).

We also evaluated the relationship between the different genotypes and serum levels of cytokines (**Supplementary Table 3**). There is no significant correlation between the genotype and serum concetration of IL-13. A significant correlation was found between the IL-1α rs1800587 genotype and its cytokine concentrations. Patients with the AA genotype have a significantly higher IL-1α cytokine concentration than those with the GG genotype (P=0.027) or the GA genotype (P=0.024). The cytokine concentration of TNF-α rs1799964 genotypes is also significantly different (P<0.001).

### Relationship Among Cytokine Gene Polymorphism, Cytokine Serum Level, and Clinical Characteristics

The possible association between cytokine serum level and polymorphic cytokine genotype of the patients was analyzed, respectively. Factors including age, gender, duration before treatment, percentage of eosinophils, number of eosinophils in whole blood, whether hospitalized, mucosa involvement, whether the lesion is extensive, whether complications occurred, DIF, and IIF results were compared. IL-13 is the only cytokine that showed a significant difference. We found that gender is significantly correlated with IL-13 rs20541 genotype (P = 0.027), and the ratio of AG in female is higher than that in male (AG & AA: P = 0.036, AG & GG: P = 0.073). Gender is significantly correlated with the rs1800925 genotype (P=0.030), and females had a higher frequency of CC genotypes than males (CC & TT: P=0.041, CC & CT: P=0.092). Moreover, the serum concentration level of IL-13 is higher in patients with high percentage or numbers of eosinophils, although without significant difference (P=0.082) (**Table 3**).

**Table 3:** Correlation of the IL-13 genotypes and IL-13 serum levels with clinical characteristics and demographic data in BP patients.

Characteristics		IL-13 rs20541				*IL-13 rs1800925*				*Serum IL-13 pg/mL*	
		GG	AG	AA	*P^1^*	CC	CT	TT	*P*	mean ± SD	*P*
Age	>60	8	11	2	0.953	13	6	3	0.313	132.67 ± 299.21	0.402
	≤60	6	8	1		11	4	0		54.89 ± 56.55	
Gender	female	6	14	0	0.027*	17	4	0	0.030*	154.74 ± 324.86	0.138
	male	8	5	3		7	6	3		44.73 ± 27.09	
Duration before treatment	>5 months	5	6	2	0.501	10	3	1	0.804	98.18 ± 168.97	0.823
	≤5 months	9	13	1		14	7	2		117.48 ± 307.39	
EOS%	>5%	6	9	1	0.943	12	5	0	0.264	31.37 ± 9.13	0.082
	≤5%	6	7	1		9	3	2		174.03 ± 337.68	
EOS	>0.5×109/L	7	10	1	0.848	14	5	0	0.147	31.01 ± 9.48	0.082
	≤0.5×109/L	5	5	1		6	3	2		174.03 ± 337.68	
Hospitalization	no	10	14	2	0.965	19	5	3	0.119	94.09 ± 143.72	0.646
	yes	4	5	1		5	5	0		135.1 ± 382.8	
Mucosa involvement	no	8	12	2	0.921	16	4	3	0.127	132.21 ± 294.47	0.379
	yes	6	7	1		8	6	0		48.29 ± 30.12	
Extensive lesion	no	6	9	1	0.891	11	4	2	0.719	60.09 ± 52.17	0.259
>50% area	yes	8	10	2		13	6	1		152.58 ± 342.1	
Complications	no	13	17	3	0.811	22	9	3	0.855	115.29 ± 266.86	0.674
	yes	1	2	0		2	1	0		57.82 ± 49.65	
DIF IgG	–	10	10	2	0.538	15	5	3	0.293	118.38 ± 298.19	0.794
	+	4	9	1		9	5	0		95.64 ± 178.08	
DIF C3	–	10	11	1	0.431	16	5	2	0.65	141.57 ± 311.76	0.308
	+	4	8	2		8	5	1		51.1 ± 41.48	
IIF	–	5	10	2	0.491	10	6	2	0.503	202.63 ± 391.77	0.168
	+	9	9	1		14	4	1		49.26 ± 37.98	

^1^*denotes P<0.05. The IL-13 genotypes with alleles (GG, AG, AA for rs20541 and CC, CT, TT for rs1800925) were calculated and associated with BP patients’ data. A significant association with gender was found in the IL-13 rs20541 (*P*=0.027) and rs1800925 (*P*=0.030).

In the Kaplan-Meier analysis for the relationship between cytokines and BP’s recurrence (**Supplementary Figure 2** and **Supplementary Table 4**), IL-13 rs20541(P=0.048) and rs1800925 genotype (P<0.001) were significantly correlated with recurrence. For IL-13 rs20541, the ratio of AG in relapsed patients was significantly lower than that of AA genotype (P=0.025); for IL-13 rs1800925, the ratio of rs1800925 TT genotype in relapsed patients was higher than that of CC and CT genotype (CC &TT, P<0.001, CT & TT, P=0.025).

## Discussion

**Figure 2:**
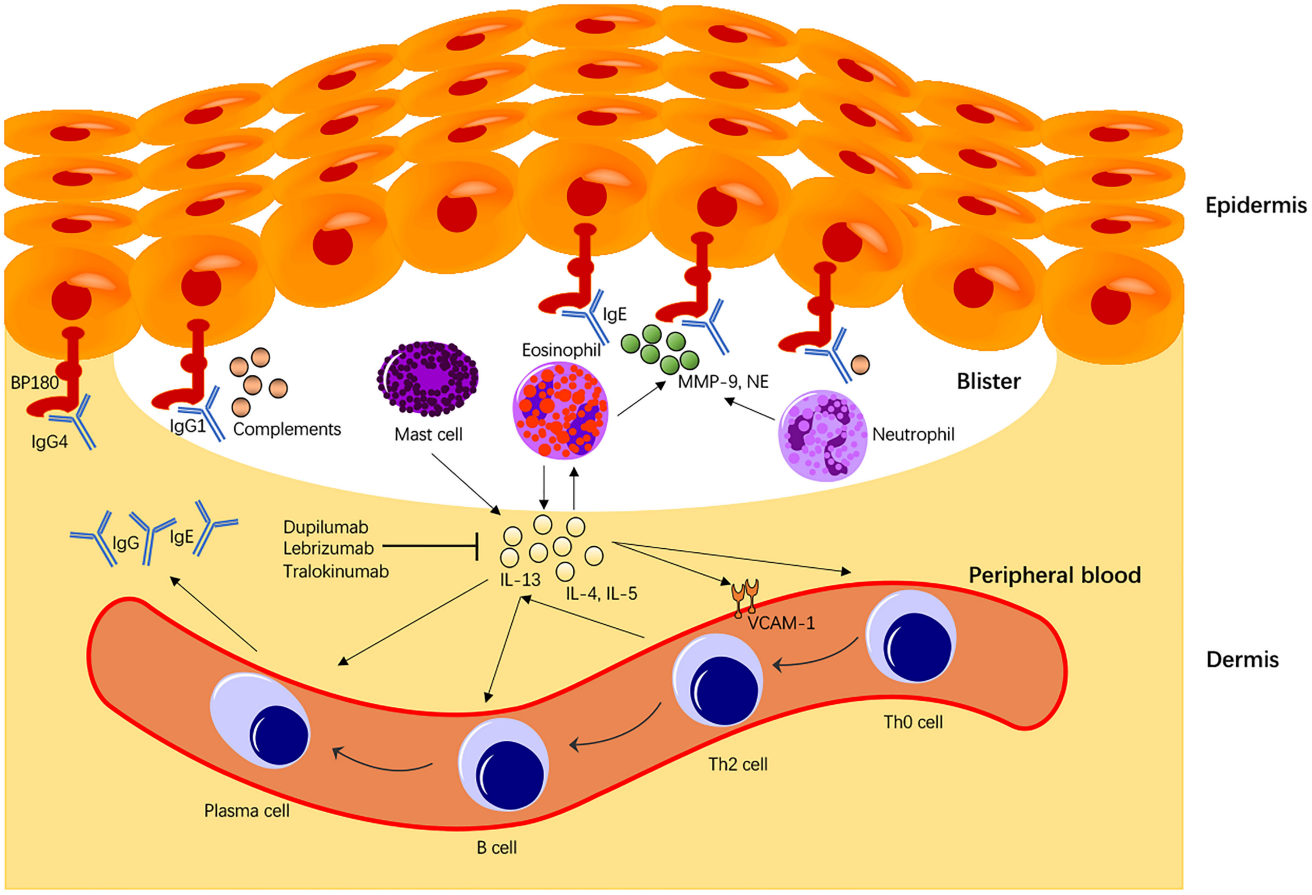
Immunological roles of IL-13 in BP pathogenesis. IL-13 secreted from Th2 cells, or mast cells activates eosinophils that produce more IL-13 molecules in a “positive feedback” pattern. IL-13 also mediates the transition of Th0 to Th2 cells, the primary cells expressing IL-13. Moreover, IL-13 promotes activated B cell maturation to plasma cells expressing BP IgE autoantibodies and enhances BP immunoglobulins (Igs) class switching. IL-13 also enhances VCAM-1 expression that promotes transmigration of eosinophils and basophils to the dermis. In BP, IL-13 also promotes B cell maturation and directly stimulates plasma cells to secrete BP autoantibodies that play a central role in the BP disease pathomechanism. Secretion of proteolytic enzymes including NE and Metalloproteinase-9 from eosinophils or neutrophils causes degradation of BP180 molecules, contributing to the BP disease pathology.

The current study revealed that IL-13 is correlated with BP in alleles, genotype, haplotype, and serum cytokine concentration. IL-13 is predominantly secreted by Th2 cells, although mast cells found in the inflammatory infiltrates of BP also produce bioactive IL-13 *in vitro* (29). IL-13 up-regulates gene expression VCAM-1, a cell adhesion molecule and marker of endothelial activation marker (30). VCAM-1 is a crucial mediator for transmigration of eosinophils and basophils expressing the ligand VLA-4 for VCAM-1. Additionally, IL-13 exerts profibrotic and proinflammatory effects on the human conjunctival fibroblasts by up-regulating the expression of the T cell co-stimulatory molecules CD80, CD40, and CD154, suggesting a significant role in the pathophysiology in mucous membrane pemphigoid (MMP) (31). IL-13 activates eosinophils to secrete various cytokines (including IL-13), promote B cell maturation into plasma cells, and mediate plasma cells to secrete IgE antibodies. Moreover, IL-13 can also transform Th0 cells into Th2 cells to maintain and enhance the entire autoimmune process (**Figure 2**). In BP, IgE titer was reported to be correlated with disease activity (32). The pathogenic effect of BP180-specific IgE auto antibodies was mediated through the degranulation of eosinophils and mast cells (32, 33). IgE antibodies could also regulate Th2 cells through IL-4 and IL-13 stimulation (34), contributing to a further loss of tolerance against BP180 antigen. Th2 cells produce cytokines such as IL-4, IL-5, IL-13, and other chemokines (including eosinophil chemotactic factor and single-cell chemotactic protein), which are overexpressed in damaged BP skin in the early stage of the disease (35). Interestingly, eosinophils could participate in maintaining Th2-type responses by producing IL-4 and IL-13 (34). This is consistent with our finding that serum IL-13 concentration is positively correlated with the number of eosinophils, which produce even more IL-13 and further activate eosinophils in a “positive feedback” pattern.

One of our major findings is that the serum IL-13 level in our BP patients was significantly higher than that in healthy subjects, supporting that IL-13 is implicated in the pathomechanism of BP *via* the Th2 cell-mediated immune response and eosinophil activation (**Figure 2**). Consistent with this finding, CD3+CD4+ or CD3+CD8+ T cells expressing IL-13 and several other cytokines from the peripheral blood were found significantly higher in BP than those from healthy subjects (20). Interestingly, the percentage of cutaneous lymphocyte-associated antigen (CLA) positive cells expressing IL-13 (CLA+IL-13+) was significantly higher than that of peripheral CLA-IL-13+ cells. Conversely, treatment of BP patients with systemic steroids significantly reduced the frequency of CLA+IL-13+ T cells. CLA is considered as the marker for skin-homing memory T cells that will selectively accumulate into the skin. Similar results were shown in ocular MMP, where treatment with immunosuppressants led to a significant reduction of CD3+ IL-13+ cells in human conjunctival fibroblasts (36), suggesting that IL-13 plays a crucial role in the profibrotic and proinflammatory responses in BP and MMP. The IL-13 monoclonal antibodies (e.g., dupilumab, lebrikizumab, and tralokinumab) have been developed for not only allergic diseases (e.g., atopic dermatitis, asthma) (37) but various other autoimmune diseases (38). The monoclonal antibody therapy (dupilumab) with dual antagonistic effects on IL-4 and IL-13 has been trialed in pilot studies for BP treatment. A patient with severe and refractory BP disease was successfully treated with dupilumab, with no recurrence ten months after remission (38). A multicenter case meta-analysis of 13 patients reported that 92.3% of BP patients treated with dupilumab led to disease remission, and 53.8% of patients achieved complete remission, with no evident adverse reaction observed in those patients (39). In the treatment of BP, targeted therapy has achieved relieving pain or itching and preventing bullae formation. However, the optimal dose, treatment interval, and election of the treatment populations remain to be established in clinical trials.

Notably, we found that IL-1β is also significantly higher in the BP patients’ sera than in the control group, consistent with a previous study (17). This could be due to IL-1β gene polymorphisms linked to BP in a Chinese population (27). IL-1 is a key mediator of innate immunity and plays a central role in many human autoinflammatory diseases. Ameglio et al. have reported a positive correlation between the number of skin lesions in BP patients and the IL-1β level in the blister fluid (18). In our study, the IL-1 SNP polymorphism showed a positive correlation with the serum level of cytokines in BP patients (**Supplementary Table 3**): The IL-1α cytokine concentration in patients with IL-1α rs1800587 AA genotype was higher than that of GG genotype (P=0.027) and GA genotype (P=0.024). IL-1 could play an essential role in BP development by inducing the synthesis and proteolytic enzyme activity of matrix metalloproteinase 9 in eosinophils and neutrophils (40, 41). The inflammatory process is initiated, further perpetuated, and enhanced by IL-1α and IL-1β (42).

Like IL-13, IL-4 is another crucial cytokine of Type 2 inflammation in which Th2 lymphocytes are the central cells of the adaptive immune response. However, we found that IL-4 is not significantly increased in sera of BP patients. Several published studies have demonstrated an elevated expression of IL-4 and IL-13 in the mononuclear cells in the skin tissue of patients with BP by immunohistochemistry or *in situ* hybridization (20, 21, 43), suggesting that IL-4 could have a pathogenic effect in the local, lesional skin. Both IL-4 and IL-13 regulate growth, survival, maturation, and activation of eosinophils (44) that contribute to the pathogenesis of BP (45). Additionally, several lines of evidence have shown that IL-4 and IL-13 could impair skin barrier function by suppressing the expression of tight junction proteins, filaggrin, loricrin, and involucrin (46–48). Other groups and we have previously shown a high incidence rate of local skin or systemic infections in BP (49–51). It will be interesting to investigate in the future whether these cytokines are involved in infections in BP patients. 

Our further analysis on alleles and genotypes showed that BP patients were more likely to carry the IL-13 rs20541 allele A. IL-13 rs20541 genotypes in BP patients were significantly different from that of the control group, with a significantly higher proportion of AG genotype. However, a recent study has shown the IL-13 rs20541 variation could play a protective role in BP (28). The discrepancy could be derived from ethnicity differences, as differences in BP incidence and age of onset between ethnic groups have been documented (52). IL-13 rs20541 is located in the IL-13 gene on chr5q31 and is a common coding SNP in exon 4. Interestingly the polymorphism of this locus has been previously associated with several human diseases such as allergic rhinitis, asthma, eczema, and psoriatic arthritis in different ethnic groups (53–56). Our study also has shown a higher frequency of C-allele in IL-13 gene variation (rs1800925) in the healthy subjects than BP patients, although not statistically significant (*P*=0.078), suggesting this C allele, instead of T allele, could have a protective effect on the susceptibility to BP (28). Our finding that BP patients were likely to carry the IL-13 rs1800925 allele T supports that IL-13 gene polymorphisms might increase BP susceptibility. The IL-13 rs1800925 gene locates on chr5q31, which may cause a C to T transition at the 1112/1024/1055 sites. The C to T transition is also related to many other diseases, including asthma, eczema, psoriatic arthritis, and breast cancer (54–57). Furthermore, we found that the CAC haplotype in IL-13 may increase the susceptibility of BP on the haplotype analysis. Collectively, our results supports that IL-13 gene polymorphism is correlated with a higher risk of BP.

While many other clinical features appear not correlated with BP gene polymorphisms, we found that gender is significantly associated with the alleles of rs20541 and rs1800925, suggesting there is a difference in genetic predisposing factors between male and female patients. As mentioned previously, a published series with a Chinese population has shown a significant association of IL-1 gene alleles (-511T and -31C) with BP in female patients (27).

The expression level of several cytokines has been correlated with BP severity and disease activity (20, 22, 30). Conversely, BP treatment with immunosuppressants could improve BP severity and reduce the cytokine-producing T cells in the skin of BP or MMP (20, 31). More recently, the number of IL-13 expressing cells has been significantly associated with itch severity (58), further supporting that IL-13 is involved in BP pathophysiology. Our analysis of the correlation between IL-13 and recurrence found that both the rs20541 and rs1800925 genotypes significantly correlate with recurrence, suggesting that patients with some genotypes be genetically more susceptible. Relapse in BP remained a persistent issue for patients and clinicians and has contributed substantially to the morbidity and mortality from BP (36). Because high IL-13 expression and gene polymorphisms are associated with BP and clinical relapse of BP, we hypothesize that IL-13 gene polymorphisms have an effect on IL-13 gene expression. However, we found no significant correlation between serum IL-13 level and IL-13 gene polymorphism, highlighting that multiple other factors such as gender may participate and act synergistically in the regulation of IL-13 expression.

Limitations of our study could be the limited number of patients and samples obtained from a single medical center. The heterogeneity of patient treatment and disease status at the time of sample collection could further introduce biases to our conclusion. Moreover, we did not further investigate how gene polymorphisms may directly affect gene expression and disease pathogenesis in BP. Furthermore, it will be interesting to investigate whether there is a significantly elevated expression of the cytokines in the perilesional and lesional skin in future work. Nevertheless, our study presented a series of patients matched with a group of healthy subjects and whose clinical features align with those previously reported series, including characteristics such as age, sex, mucosal involvement, corticosteroid dose, and relapse rate (28, 36, 51, 59–61). Remarkably, our studies have identified that BP is associated with IL-13 gene polymorphism and an increased IL-13 in the peripheral blood. Our preliminary observation also supports that the IL-13 genotypes could be valuable in predicting disease relapse of BP. Future studies with a larger sample size in a multicenter research scenario will be required to validate our results.

## Methods

### Study Population and Design

The medical records of Chinese BP patients from the Peking Union Medical College Hospital between 2014 and 2019 were searched. All 68 BP patients were Chinese and genetically unrelated to each other. The control group comprised 86 healthy individuals, and the groups were selected to match age and sex for both SNP and serum level analyses. Informed consent was obtained from each participant, and among all these subjects, 61 patients and 86 healthy subjects were available for whole blood collection. 56 BP patients and 52 healthy subjects were available for serum collection. The SNP and serum level of cytokines were examined in 51 BP patients. Complete medical profiles were available only in 37 patients with SNP results and 36 patients with serum level results, and 21 patients were regularly followed up for more than one year.

BP was diagnosed based on clinical presentation, subepidermal blister on skin biopsy, compatible anti-BP180 antibody titer more than 9U/L, positive indirect immunofluorescence microscopy (IIF), or positive direct immunofluorescence microscopy (DIF) according to investigational assessment guidelines (62). Whole blood serum samples of the patients were collected immediately after diagnosis of BP, and the peri-procedural assessment was performed by using the initial data analysis of patients. Samples and medical records of the healthy group were collected at their regular health check. BP patients were followed up by telephone calls or in our outpatient office for prognostic information. The definition of relapse is the appearance of at least three new lesions in one month (including blisters, urticarial plaques, or eczematous lesions) or no less than one large (>10 cm in diameter) urticarial plaque or eczematous lesion that does not heal within one week, or the extension of original lesions or daily pruritus in the adequately managed patients within disease control (36).

The serum cytokine level, cytokine polymorphism, and clinical characteristics of the patient group and the healthy group were analyzed retrospectively. The Human Research Ethics Committee of Peking Union Medical College Hospital considered and approved this study (JS-2338). Informed consent was obtained from all individual participants included in the study.

### Genotyping Genes and Measuring Serum Level Cytokines

We extracted genomic DNA from 5 mL of whole blood using a DNA isolation kit with magnetic beads (BioTeKe Corporation). The single nucleotide polymorphisms (SNPs) of cytokine genes including IL-1α (rs1800587), IL-1β (rs16944, rs1143627, rs1143634), IL-4 (rs2243250), IL-6 (rs1800795), IL-10 (rs1800896, rs1800871, rs1800872), IL-13 (rs1800925, rs20541), TNF-α (rs1799964, rs1800630, rs1799724, rs361525), IFN-γ (rs1799964, rs1800630, rs361525, rs1800629, rs4248160, rs1800750), and TGF-β1 (rs2317130, rs1800469, rs4803457) were determined by MassARRAY System analysis.

The DNA sequences containing each SNP of cytokine genes were amplified by polymerase chain reaction. The remaining deoxyribonucleoside triphosphate (dNTP) and primers were removed using Shrimp alkaline phosphatase enzyme. Subsequently, a single base extension primer, whose 3’ terminal base was close to the SNP site and which was completely complementary to the target fragment, was added so that the probe only extended one base at the SNP site. Besides, this primer had four ddNTPs instead of dNTPs so that the connected ddNTP corresponded to the allele of the SNP site. Matrix-assisted laser desorption/ionization time-of-flight mass spectrometry (MALDI-TOF MS) was used to detect the molecular weight difference between the extended product and the unextended primer and determine the specific base.

Serum samples were tested using the ELISA kits (human IL-1α IL-1β, IL-4, IL-6, IL-10, IL-13, TNF-α, IFN-γ, and TGF-β1 ELISA kits by Neobioscience Company) for cytokine concentrations.

### Statistical Analysis

SPSS statistical software program (version 26.0) and GraphPad Prism 8 software were used for statistical analysis in this study. The difference of cytokine serum levels between groups was assessed using the Student’s t-test or Mann-Whitney U test (2 groups) or one-way analysis of variance test (ANOVA, or Kruskal-Wallis test, more than 2 groups). During the analysis of SNPs, the Hardy-Weinberg equilibrium test was conducted with the Chi-square (χ2) test. Genotype distribution, allele frequency, linkage disequilibrium (LD), and haplotype analysis were calculated by SHEsis online software (http://analysis.biox.cn/myAnalysis.php). The χ2 test (or Fisher’s exact) is used to determine the relationship between SNP genotypes, BP occurrence, genotypes of SNP, and clinical characteristics of patients. Kaplan-Meier analysis was performed to analyze the related factors of recurrence. P<0.05 was considered to have a significant difference.

## Data Availability Statement

The original contributions presented in the study are included in the article/**Supplementary Material**. Further inquiries can be directed to the corresponding author.

## Ethics Statement

The studies involving human participants were reviewed and approved by Human Research Ethics Committee of Peking Union Medical College Hospital. The patients/participants provided their written informed consent to participate in this study.

## Author Contributions

All authors contributed to either conceptualization. Methodology: YW, XM, and LL. Software: YW and XM. Validation: YL and YY. Formal analysis: YW. Investigation: YW, YL, and YY. Resources: LL. Data curation: YW and XM. Writing—original draft preparation: YW. Writing—review and editing: XM and LL. Visualization: YW. Supervision: LL and HJ. Project administration: HJ and LL. Funding acquisition: LL. All authors have read and agreed to the published version of the manuscript.

## Funding

This work was supported by the National Natural Science Foundation of China (81972945), the National Key Research and Development Program of China Grant No. 2016YFC0901500, Milstein Medical Asian American Partnership Foundation, and Education Reform Projects of Peking Union Medical College (2016zlgc0106).

## Conflict of Interest

The authors declare that the research was conducted in the absence of any commercial or financial relationships that could be construed as a potential conflict of interest.

## Publisher’s Note

All claims expressed in this article are solely those of the authors and do not necessarily represent those of their affiliated organizations, or those of the publisher, the editors and the reviewers. Any product that may be evaluated in this article, or claim that may be made by its manufacturer, is not guaranteed or endorsed by the publisher.
