# Supplementary material for: IL-13 Genetic Susceptibility to Bullous Pemphigoid: A Potential Target for Treatment and a Prognostic Marker
Source: Front Immunol. 2022 Jan 24;13:824110. doi: 10.3389/fimmu.2022.824110 (PMC8818855; doi:10.3389/fimmu.2022.824110)
Supplement: Supplementary file 1 [file DataSheet_1.pdf]

Supplementary Figure 1. Linkage disequilibrium pattern of the genomic region in chromosome (chr) 2 (IL-1), chr5 (IL-4 & IL-13), chr1 (IL-10), chr6 (TNF- α), chr12 (IFN- γ), chr19 (TGF- β)

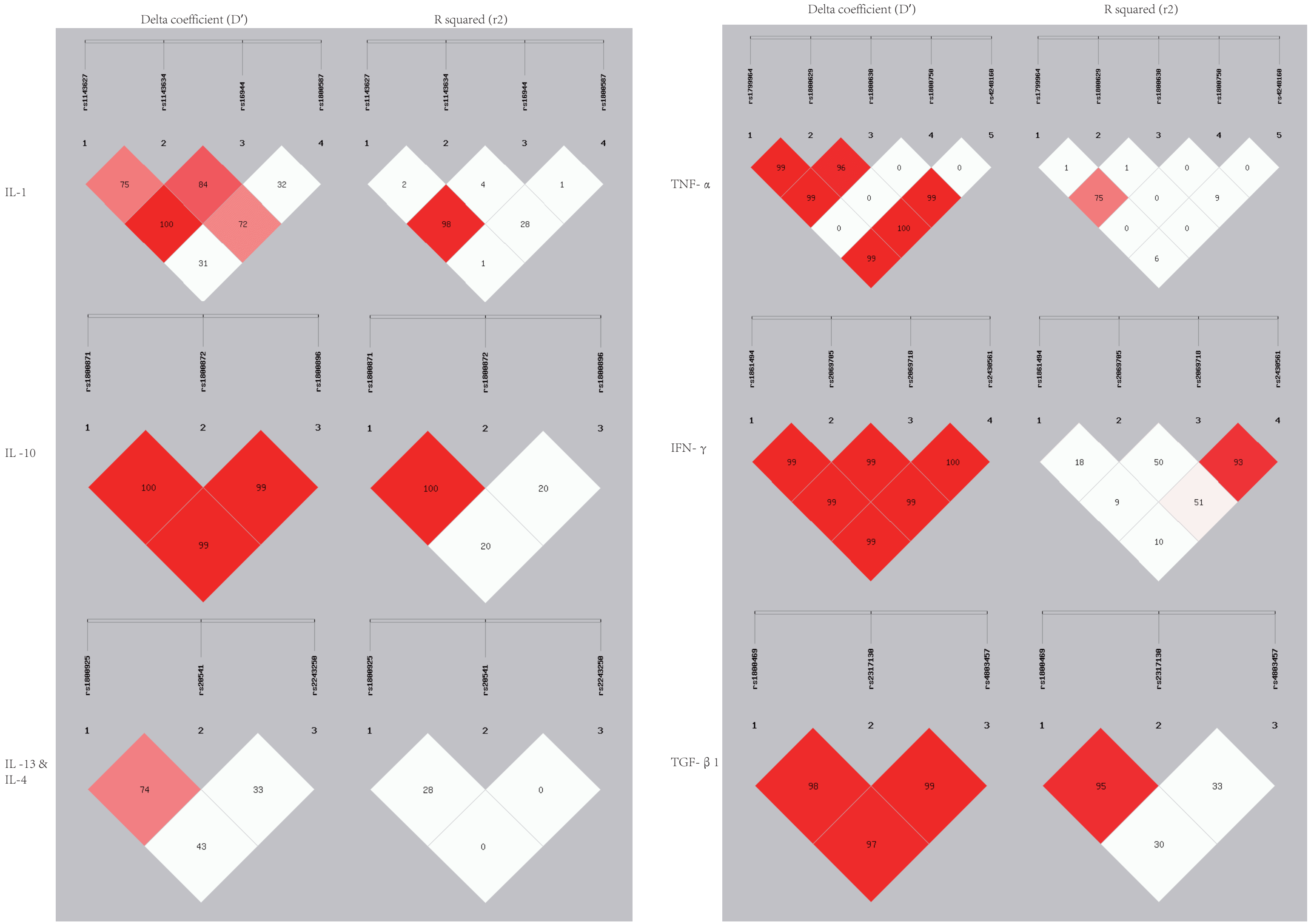

Supplementary Figure 2. Time-course curve based on Kaplan-Meier analysis to show the relationship between IL-13 and relapse.

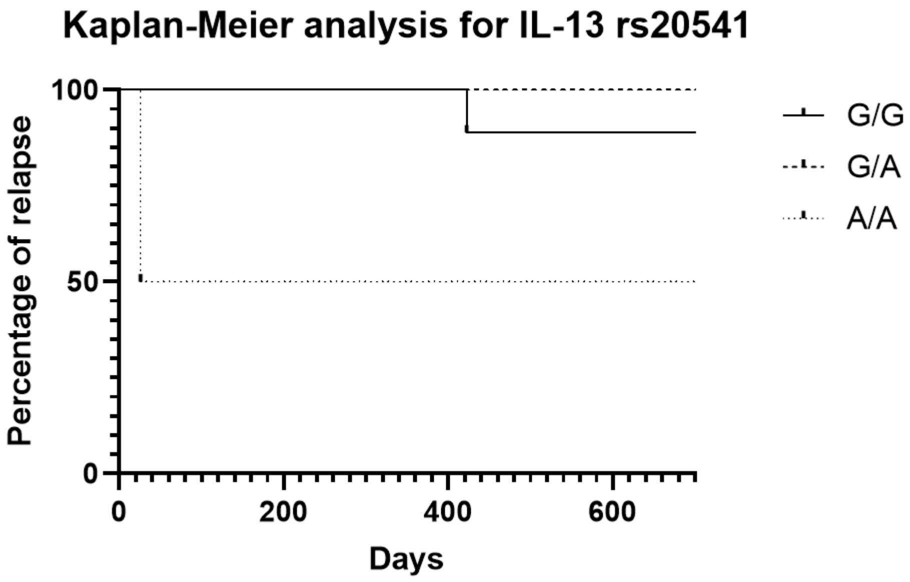

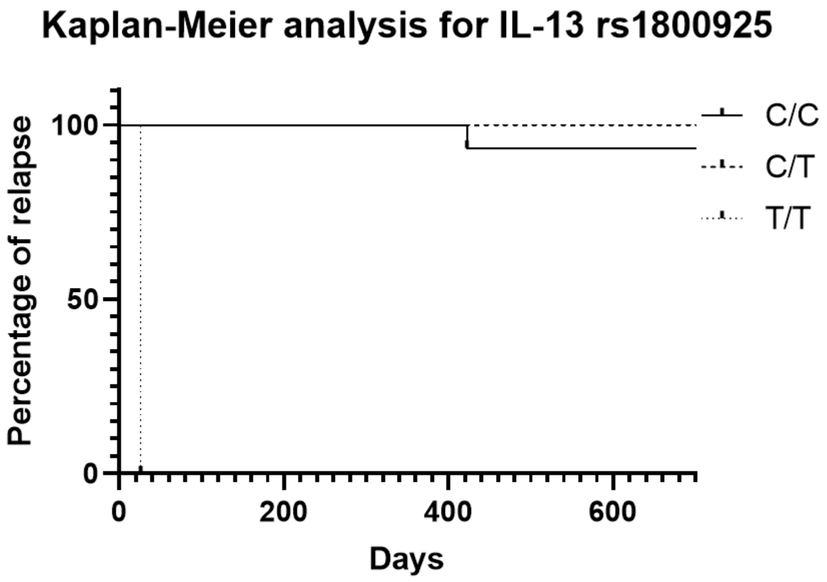

Supplementary Table 1. Genotype and allele frequencies of gene polymorphisms in Chinese patients with BP and respective controls.

Gene	Genotype/Allele	Patients	Control subjects	χ2/OR(95% CI)	P Value
rs1800587	GG	49	67		
	AG	10	16	2.884	0.236
	AA	2	0		
	G	108	150	0.823 (0.385-1.757)	0.614
	A	14	16		
rs16944	GG	19	28		
	AG	27	40	0.275	0.872
	AA	15	18		
	G	65	96	0.903 (0.567-1.439)	0.667
	A	57	76		
rs1143627	AA	17	24		
	AG	27	35	0.12	0.942
	GG	15	18		
	A	61	83	0.915(0.566-1.480)	0.719
	G	57	71		
rs1143634	GG	55	76		
	AG	4	7	0.137	0.934
	AA	2	3		
	G	114	159	1.165(0.468-2.903)	0.743
	A	8	13		
rs2243250	TT	42	53		
	CT	16	24	0.296	0.862
	CC	3	5		

	T	100	130	1.189(0.655-2.158)	0.569
	C	22	34		
rs1800795	GG	60	85		
	CG	0	0	0	1
	CC	0	0		
	G	120	170	—	1
	C	0	0		
rs1800896	TT	49	65		
	CT	11	18	0.242	0.623
	CC	0	0		
	T	109	148	1.205(0.547-2.655)	0.643
	C	11	18		
rs1800871	AA	29	35		
	AG	19	33	1.545	0.462
	GG	13	13		
	A	77	103	0.980(0.602-1.596)	0.936
	G	45	59		
rs1800872	TT	29	35		
	GT	19	33	2.012	0.57
	GG	12	13		
	T	77	103	1.026(0.627-1.677)	0.919
	G	43	59		
rs1799964	TT	43	60		
	CT	14	18	0.315	0.854
	CC	3	6		
	T	100	138	1.087(0.584-2.024)	0.793
	C	20	30		

rs1800630	CC	48	63		
	AC	9	17	0.809	0.667
	AA	2	4		
	C	105	143	1.412(0.690-2.889)	0.343
	A	13	25		
rs361525	GG	55	80		
	AG	5	5	0.329	0.566
	AA	0	0		
	G	115	165	0.697(0.197-2.463)	0.741
	A	5	5		
rs1800629	GG	53	70		
	AG	7	12	0.992	0.609
	AA	0	1		
	G	113	152	1.487(0.581-3.804)	0.405
	A	7	14		
rs4248160	GG	59	83		
	AG	2	0	3.496	0.174
	AA	0	1		
	G	120	166	0.723(0.100-5.204)	0.746
	A	2	2		
rs1800750	GG	60	83		
	AG	0	0	0	1
	AA	0	0		
	G	120	166	—	1
	A	0	0		
rs2069705	GG	39	50	0.35	0.84
	AG	18	27		

	AA	4	4		
	G	96	127	1.018(0.574-1.804)	0.952
	A	26	35		
rs1861494	TT	24	25		
	CT	26	39	0.915	0.633
	CC	11	14		
	T	74	89	1.161(0.717-1.880)	0.545
	C	48	67		
rs2069718	AA	48	63		
	AG	10	19	1.556	0.669
	GG	2	2		
	A	106	145	1.201(0.590-2.443)	0.613
	G	14	23		
rs2317130	TT	16	22		
	CT	34	38	1.245	0.537
	CC	11	20		
	T	66	82	1.121(0.699-1.798)	0.635
	C	56	78		
rs1800469	TT	16	22		
	CT	35	43	2.001	0.368
	CC	9	21		
	T	67	87	1.235(0.773-1.972)	0.376
	C	53	85		

Supplementary Table 2. Haplotype patterns with their frequencies in the population.

	Haplotype	Case (frequncy)	Control (frequncy)	Chi2	Fisher's P	Pearson's P	Odds Ratio (95%CI)
IL-1	A A G A	6.00(0.051)	6.91(0.045)	0.037	0.847	0.847	1.117 (0.364-3.427)
	A A G G	0.00(0.000)	1.06(0.007)	-	-	-	-
	A G G A	1.95(0.016)	3.90(0.025)	0.271	0.603	0.603	0.633 (0.111-3.597)
	A G G G	53.05(0.450)	71.13(0.462)	0.126	0.723	0.723	0.916 (0.565-1.486)
	G A A A	0.00(0.000)	0.00(0.000)	-	-	-	-
	G A A G	0.00(0.000)	1.03(0.007)	-	-	-	-
	G G A A	6.05(0.051)	4.19(0.027)	0.999	0.318	0.318	1.892 (0.532-6.733)
	G G A G	50.95(0.432)	64.78(0.421)	0.002	0.967	0.967	1.010 (0.621-1.643)
	G G G G	0.00(0.000)	1.00(0.006)	-	-	-	-
IL-10	A T T	77.00(0.642)	100.00(0.633)	0.023	0.881	0.881	1.039 (0.634-1.702)
	G G C	11.00(0.092)	18.00(0.114)	0.362	0.548	0.548	0.785 (0.356-1.731)
	G G T	32.00(0.267)	40.00(0.253)	0.065	0.799	0.799	1.073 (0.625-1.842)
	A T C	0.00(0.000)	0.00(0.000)	-	-	-	-
TNF-α	C A A G G	0.00(0.000)	1.27(0.008)	-	-	-	-
	C G A G A	2.00(0.017)	2.00(0.012)	0.109	0.742	0.742	1.392 (0.193-10.030)
	C G A G G	11.00(0.095)	19.73(0.122)	0.532	0.466	0.466	0.749 (0.343-1.633)
	C G C G G	5.00(0.043)	5.00(0.031)	0.278	0.598	0.598	1.403 (0.397-4.962)
	T A C G G	7.00(0.060)	10.73(0.066)	0.046	0.83	0.83	0.898 (0.336-2.401)
	T G C G G	91.00(0.784)	123.27(0.761)	0.119	0.731	0.731	1.106 (0.623-1.964)
IFN-γ	C A G A	0.00(0.000)	0.00(0.000)	-	-	-	-
	C G A T	48.00(0.400)	67.00(0.429)	0.243	0.622	0.622	0.886 (0.546-1.436)
	T A A T	10.00(0.083)	13.00(0.083)	0	1	1	1.000 (0.423-2.366)

	T A G A	14.00(0.117)	19.00(0.122)	0.017	0.897	0.897	0.952 (0.456-1.987)
	T G A T	48.00(0.400)	57.00(0.365)	0.344	0.557	0.557	1.158 (0.710-1.889)
TGF-β1	A C T	49.00(0.430)	76.00(0.481)	0.755	0.385	0.385	0.806 (0.495-1.311)
	A T C	0.00(0.000)	1.00(0.006)	-	-	-	-
	G C T	1.00(0.009)	1.00(0.006)	-	-	-	-
	G T C	31.00(0.272)	43.00(0.272)	0.001	0.981	0.981	0.993 (0.578-1.709)
	G T T	33.00(0.289)	37.00(0.234)	1.024	0.312	0.311	1.327 (0.767-2.295)

Supplementary Table 3. Comparison of cytokine's serum levels among patients with different genotype.

Cytokines	SNP	Genotype	mean ± SD (pg/mL)	P	Genotype	mean ± SD (pg/mL)	P	Genotype	mean ± SD (pg/mL)	P
IL-1α	rs1800587	GG	11.01 ± 33.93	0.566	GG	11.01 ± 33.93	0.027**	GA	2.28 ± 2.87	0.024**
		GA	2.28 ± 2.87		AA	68.14 ± 95.79		AA	68.14 ± 95.79	
IL-1β	rs16944	GG	13.68 ± 15.12	0.122	GG	13.68 ± 15.12	0.9	GA	75.67 ± 178.31	0.122
		GA	75.67 ± 178.31		AA	7.82 ± 10.43		AA	7.82 ± 10.43	
	rs1143627	AA	12.61 ± 15.54	0.139	AA	12.61 ± 15.54	0.922	AG	75.67 ± 178.31	0.13
		AG	75.67 ± 178.31		GG	7.82 ± 10.43		GG	7.82 ± 10.43	
	rs1143634	GG	43.13 ± 129.28	0.641	GG	43.13 ± 129.28	0.82	GA	8.13 ± 14.08	0.901
		GA	8.13 ± 14.08		AA	22.47 ± 14.72		AA	22.47 ± 14.72	
IL-4	rs2243250	TT	4.55 ± 6.57	0.22	TT	4.55 ± 6.57	0.869	CT	8.28 ± 14.71	0.495
		CT	8.28 ± 14.71		CC	3.44 ± 3.89		CC	3.44 ± 3.89	
IL-6	rs1800795	GG	22.59 ± 76.25	-	GG		-	GC		-
		GC			CC			CC		
IL-10	rs1800896	TT	0.92 ± 1.87	0.107	TT		-	CT		-
		CT	3.25 ± 8.44		CC			CC		
	rs1800871	AA	0.93 ± 1.82	0.951	AA	0.93 ± 1.82	0.261	AG	1.01 ± 2.22	0.353
		AG	1.01 ± 2.22		GG	2.44 ± 7		GG	2.44 ± 7	
	rs1800872	TT	0.93 ± 1.82	0.952	TT	0.93 ± 1.82	0.241	GT	1.01 ± 2.22	0.952
		GT	1.01 ± 2.22		GG	2.57 ± 7.3		GG	2.57 ± 7.3	
IL-13	rs1800925	CC	117.21 ± 262.82	0.357	CC	117.21 ± 262.82	0.647	CT	47.22 ± 47.5	0.958
		CT	47.22 ± 47.5		TT	54.86 ± 15.05		TT	54.86 ± 15.05	
	rs20541	GG	64.15 ± 92.62	0.29	GG	64.15 ± 92.62	0.872	AG	134.77 ± 313.74	0.46
		AG	134.77 ± 313.74		AA	44.42 ± 22.95		AA	44.42 ± 22.95	
TNF-α	rs1799964	TT	50.24 ± 106.53	0.937	TT	50.24 ± 106.53		TC	58.06 ± 63.84	

		TC	58.06 ± 63.84		CC	1371.02 ± 1858.47	9.0212E-8***	CC	1371.02 ± 1858.47	3.4718E-7***
	rs1800630	CC	50.08 ± 100.74	0.601	CC		-	CA		-
		CA	70.98 ± 69.13		AA			AA		
	rs361525	GG	110.81 ± 405.08	0.743	GG		-	GA		-
		GA	50.6 ± 43.48		AA			AA		
	rs1800629	GG	114.6 ± 404.52	0.594	GG		-	GA		-
		GA	16.57 ± 13.76		AA			AA		
	rs4248160	GG	106.42 ± 388.31	0.802	GG		-	AG		-
		AG	36.54 ± 12.4		AA			AA		
IFN-γ	rs2069705	GG	56.52 ± 156.73	0.881	GG	56.52 ± 156.73	0.856	GA	49.98 ± 120.26	0.929
		GA	49.98 ± 120.26		AA	42.86 ± 38.84		AA	42.86 ± 38.84	
	rs1861494	TT	52.28 ± 104.57	0.506	TT	52.28 ± 104.57	0.145	TC	24.47 ± 22.11	0.053
		TC	24.47 ± 22.11		CC	135.86 ± 306.49		CC	135.86 ± 306.49	
	rs2069718	AA	49.13 ± 140.19	0.672	AA	49.13 ± 140.19	0.945	GA	71.55 ± 160.45	0.791
		GA	71.55 ± 160.45		GG	41.91 ± 44.43		GG	41.91 ± 44.43	
	rs2430561	TT	49.13 ± 140.19	0.669	TT	49.13 ± 140.19	0.95	TA	71.55 ± 160.45	0.856
		TA	71.55 ± 160.45		AA	54.45 ± 38.19		AA	54.45 ± 38.19	
TGF-β1	rs2317130	TT	995.28 ± 496.11	0.385	TT	995.28 ± 496.11	0.803	TC	848.72 ± 302.98	0.61
		TC	848.72 ± 302.98		CC	1040.16 ± 315.06		CC	1040.16 ± 315.06	
	rs4803457	TT		-	TT	902.42 ± 322.19	0.389	TC		-
		TC			CC	1011.7 ± 514.47		CC		
	rs1800469	GG	995.28 ± 496.11	0.348	GG	995.28 ± 496.11	0.967	GA	877.41 ± 335.23	0.459
		GA	877.41 ± 335.23		AA	988.27 ± 242.26		AA	988.27 ± 242.26	

Supplementary Table 4. Survival analysis for the relationship between the relapse and IL-13 genotypes. A significant correlation was found between the IL-13 SNPs rs20541(P=0.048) or rs1800925 (P<0.001) and the relapse rate in the BP patients, respectively.

IL-13 SNPs	Genotype	Relapse	Relapse Free	P-Value[1]
rs20541	G/G	1	8	0.048*
	A/G	0	10	
	A/A	1	1	
rs1800925	C/C	1	14	0.000042**
	C/T	0	5	
	T/T	1	0	

1*denotes P<0.05, **denotes P<0.01.
